# Does a training course on early childhood communication have an impact on the knowledge of early childhood education professionals?

**DOI:** 10.1590/2317-1782/20242023192en

**Published:** 2024-06-14

**Authors:** Hellen Tatyanne da Silva Barbosa, Vanessa Giacchini, Eliene Silva Araújo, Ana Manhani Cáceres-Assenço

**Affiliations:** 1 Laboratório de Desenvolvimento da Linguagem, Departamento de Fonoaudiologia, Centro de Ciências da Saúde, Universidade Federal do Rio Grande do Norte – UFRN - Natal (RN), Brasil.

**Keywords:** Training, Child Education, Retention, Communication Language Development, Communication Disorders

## Abstract

**Purpose:**

To analyze whether a training course on communication development in early childhood has a positive impact on the knowledge of early childhood education professionals and to verify the participants' perception of the course.

**Methods:**

A longitudinal study conducted in a virtual environment between September 2021 and December 2022. A total of 91 early childhood education professionals took part and completed a training course. The course consisted of three modules on communication development in early childhood, offered through the Google Classroom platform, with a total workload of 50 hours spread over four months. Participants answered a questionnaire made up of 20 items related to the topics covered before starting the course, immediately after completing it and six months after finishing. For each question answered correctly, 1 point was awarded. The data was analyzed using a descriptive and inferential approach, and the total number of correct answers at the three moments was compared using Friedman's ANOVA, with a significance level of 5%.

**Results:**

Both the analysis of the correct answers to each item in the questionnaire and the overall score showed a gradual increase between the three moments. The participants' perception of the course was highly satisfied.

**Conclusion:**

The participants showed an increase in the number of correct answers to the questionnaire before and after the training course, which suggests greater knowledge about the development of communication in early childhood both immediately after the course and after six months.

## INTRODUCTION

Speech-Language Pathology began to develop in the 1920s. At that time, those performing activities similar to those of modern speech-language pathologists had training related to education, highlighting a connection between the profession and teaching^(1)^. It was not until the 1980s, with the regulation of the profession, that schools were legally recognized as workplaces for speech-language pathologists. Today, Speech-Language Pathology in Educational Environment is not limited to screenings, guidance, and referrals but also encompasses active participation in the educational process, including working with parents, teachers, students, and other professionals that comprise the school team^(2,3)^.

On the one hand, speech-language pathologists are professionals specialized in coping with communication disorders, while early childhood education professionals are essential in creating an educational environment suitable for stimulating the development of language and communication in children^(4)^. Thus, the partnership between Speech-Language Pathology and Education can be a strategic link in promoting communication development in early childhood.

During a child’s first five years of life, development occurs intensely. Emotional, social, and cognitive skills are acquired and integrated into everyday life, especially in the school context^(5)^. Thus, school is not limited to learning academic content but also facilitates the development of skills and competencies. Among these, language skills are noteworthy, as a variety of interlocutors is crucial for communicative ability^(6)^.

Outside the family, educators are the most influential agents in a child’s development. Through their daily contact, they can be the first to notice difficulties in communication and learning^(7,8)^, and the effects of their actions extend far beyond a child’s ability to read and write^(9,10)^. However, for educators to fulfill this important role, they must have a solid understanding of communication development in early childhood. In practice, many educators face difficulties in identifying and acting appropriately in situations involving child communication^(11,12)^.

With their specialized training, speech-language pathologists can contribute to the ongoing education and professional development of educators. This collaboration can help educators improve their approach to communication challenges in children, fostering a more inclusive and motivating learning environment^(13)^. To ensure that ongoing education translates into practical knowledge, it is necessary to measure its effectiveness, that is, the difference in knowledge before and after the training course. However, understanding whether this change occurs is not enough; the professional’s retention of this knowledge for an extended period after the course completion must be verified.

This study aimed to analyze whether a training course on the development of communication in early childhood positively impacts the knowledge of early childhood education professionals and to verify the participants’ perception of this course.

## METHOD

The study was approved by the Research Ethics Committee linked to Onofre Lopes University Hospital of the Federal University of Rio Grande do Norte under protocol no. 4,955,205. Participants were informed about the objectives and procedures of the research and invited to express their consent through a virtual Informed Consent Form (ICF).

Data were collected between September 2021 and December 2022. The sample was obtained for convenience from those enrolled in a training course on communication development in early childhood. This course was advertised on social media by the associated research laboratory and the University. A total of 91 education professionals who completed the training course participated in the study. The inclusion criteria were being an education professional and completing the training course. The only exclusion criterion was not working in early childhood education. Tables 1 and 2 present the sociodemographic characteristics and professional profiles of the participants. It is worth noting that a considerable number of participants work in both early childhood education and other teaching levels.

**Table 1:** Frequency distribution of the sociodemographic variables of the participants

**Variable**		**Frequency**	**%**
**Sex**	Female	86	94.5
	Male	5	5.5
**Age**	Up to 24 years	4	4.4
	25 to 29 years	15	16.5
	30 to 34 years	14	15.4
	35 to 39 years	21	23.1
	40 to 44 years	19	20.9
	45 to 49 years	11	12.1
	50 to 54 years	5	5.5
	55 years or older	2	2.2
**Highest Level of Education**	High School	1	1.1
	Bachelor's Degree - Education	19	20.9
	Bachelor's Degree - other Teaching Degrees	9	9.9
	Specialization	55	59.8
	Master's Degree (academic or professional)	3	3.3
	Ph.D.	1	1.1
	Other*	4	4.4
**Socioeconomic Classification**	A1	10	11.0
	B1	9	9.9
	B2	36	39.6
	C1	23	25.3
	C2	12	13.2
	D-E	1	1.1
**Location**	Natal	41	45.1
	Other cities in the state of Rio Grande do Norte	47	51.6
	Other states	3	3.3

* Includes professionals with a teaching degree or those currently pursuing higher education

**Table 2:** Frequency distribution of variables related to the professional activities of the participants

**Variable**		**Frequency**	**%**
**Professional Practice in Education**	1 to 3 years	17	18.7
	4 to 5 years	13	14.3
	6 to 10 years	29	31.9
	11 to 15 years	15	16.5
	16 to 20 years	10	11.0
	More than 20 years	7	7.7
**Practice in Early Childhood Education**	1 to 3 years	25	27.5
	4 to 5 years	11	12.1
	6 to 10 years	32	35.2
	11 to 15 years	10	11.0
	16 to 20 years	9	9.9
	Over 20 years	2	2.2
	Not applicable	2	2.2
**Educational Level of Engagement** *	Early Childhood Education Center (CEMEI)	51	56.0
	Daycare	37	40.7
	Elementary School I	39	42.9
	Elementary School II	8	8.8
	High School	4	4.4
	Adult and Youth Education	1	1.1
**Position**	Teacher	57	62.7
	Pedagogical Coordinator	14	15.3
	Child Development Assistant	9	9.9
	Intern	3	3.3
	Manager	3	3.3
	Pedagogical Support	1	1.1
	Specialized Educational Assistance (SEA) Teacher	1	1.1
	Multifunctional Resource Room Teacher	1	1.1
	Therapeutic Attendant	1	1.1
	Currently not practicing	1	1.1

* Given that many participants work in more than one educational institution and often at different educational levels, the number of times each level was indicated was tallied

### Context

**Figure 1:**
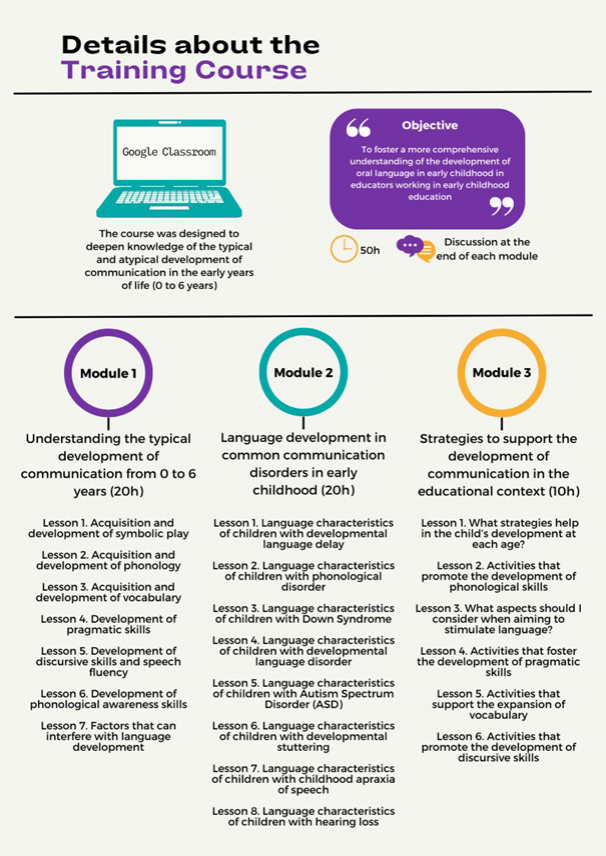
Details of the training course characteristics

The training course was conducted via the Google Classroom virtual platform, with a total duration of 50 hours spread over four months. It was developed as part of a university extension project aimed at education professionals, and its curriculum covered knowledge about typical and atypical communication development in early childhood. The content was divided into three modules: (1) Acquisition and development of oral language; (2) Common communication disorders in early childhood; (3) Strategies to stimulate communication in the educational setting. The classes were taught by university faculty from the Speech, Language and Hearing Sciences program whose area of expertise includes language or hearing in childhood. Each module consisted of video lessons of about 30 minutes each. At the end of each class, participants were required to answer five multiple-choice questions on the topic (Figure 1).

Each participant accessed the material individually and set their own pace of study, guided by pre-established deadlines. The modules were released at four-week intervals, with a total deadline of 16 weeks to complete the course. It should be highlighted that two cohorts of the course were offered during the study period, both with the same content and workload. Out of 383 individuals enrolled in the course, 203 (53.0%) accessed the lessons, and of these, 123 (32.1%) completed all activities within the deadline. Thirty-two of these had to be excluded for not meeting the exclusion criterion or for not consenting to participate.

### Outcome measures

To measure knowledge about communication development in early childhood, a questionnaire was developed based on the instrument used in other studies that assessed the efficacy of training courses for primary healthcare professionals^(14)^. We retained the structure of 20 items, the response options, and the scoring method; however, the statements were adapted to exclusively cover the contents of the training course.

When answering the questionnaire, participants were required to mark each item as “true” or “false.” Additionally, the “I don’t know” option was included to minimize the occurrence of random responses, allowing participants to indicate when they were unsure about a specific item. One point was awarded for each correctly answered question, making the score range from zero to 20.

The questionnaire was made available online via the Google Forms platform and was administered at three distinct moments: (1) before starting the course, (2) immediately after its completion, and (3) six months after the course had ended.

To assess how relevant each participant found the course content to their practice, we developed a questionnaire on their perception of the course. This questionnaire consisted of six statements to be answered on a five-point Likert scale with the following response options: strongly disagree, disagree, neutral, agree, and strongly agree. Notably, it was administered immediately after the course completion, and the analysis considered the percentage of participants who selected each response option per statement.

### Data analysis

The analysis of the knowledge questionnaire considered both the percentage of correct answers for each questionnaire item and the total percentage of correct responses per participant. To assess the level of efficacy and information retention from the training, we compared the correct answers at the three moments when the questionnaire was administered. The statistical analysis was performed using the Statistical Package for the Social Sciences (SPSS), version 24. Descriptive analysis was used to examine the frequency of correct answers, and inferential analysis was conducted using Friedman’s test with multiple comparisons at a significance level of 5%.

## RESULTS

The analysis of each item on the questionnaire revealed a gradual increase in the percentage of correct responses over the three moments, with a few exceptions (items 2, 3, 11, 16, and 17). The analysis for each time showed that before the training, three items had less than 50% correctness (items 1, 10, and 15), immediately after training only one item remained below 50% correctness (item 15), while at the later post-training moment the lowest frequency of correctness was 71.4% (Table 3).

**Table 3:** Percentage of correct responses for each item of the questionnaire on knowledge of communication development

**Item**	**Pre**	**Immediate Post**	**Late Post**
1. A child born deaf cannot develop normal oral language.	46.2	73.6	83.5
2. If a child does not speak yet, they cannot understand what they hear.	91.2	86.8	98.9
3. Symbolic play enhances language development.	100.0	98.9	100.0
4. A child with hearing problems may exhibit behavioral issues as a consequence of difficulty hearing.	87.9	91.2	95.6
5. During the first years of life, children may show symptoms of stuttering.	68.1	91.2	100.0
6. Knowing many words helps children to pay attention to the sounds that comprise them.	79.1	83.5	91.2
7. The human ear is capable of hearing low-, mid-, and high-frequency sounds.	73.6	87.9	100.0
8. It is normal for a child not to combine words at 2 years of age.	54.9	64.8	83.5
9. When a child has difficulties communicating verbally, they have less capacity to learn.	71.4	89.0	98.9
10. Every hearing-impaired child will have significant difficulty hearing what people say.	48.4	62.6	75.8
11. A child who plays with smartphones, tablets, or computers most of the day learns to speak better.	93.4	90.1	98.9
12. Rhymes and alliterations favor language development.	92.3	95.6	98.9
13. Untreated ear infections can cause hearing loss.	82.4	92.3	98.9
14. When talking to a child who is having trouble communicating, it is interesting to speak quickly and with long, well-constructed sentences.	94.5	97.8	98.9
15. Children under one year generally repeat words when asked.	37.4	39.6	71.4
16. When we ask a child something, we need to give them time to respond before helping with the answer.	96.7	96.7	100.0
17. Deaf children cannot go to school.	98.9	98.9	100.0
18. By 5 years old, a child should be able to articulate all sounds of their native language clearly.	54.9	76.9	87.9
19. A child who stutters can cause other children in their environment to start stuttering.	87.9	89.0	100.0
20. If a child speaks in a way that is difficult to understand at 4 years old, they need to undergo a speech therapy assessment.	89.0	96.7	100.0

**Figure 2:**
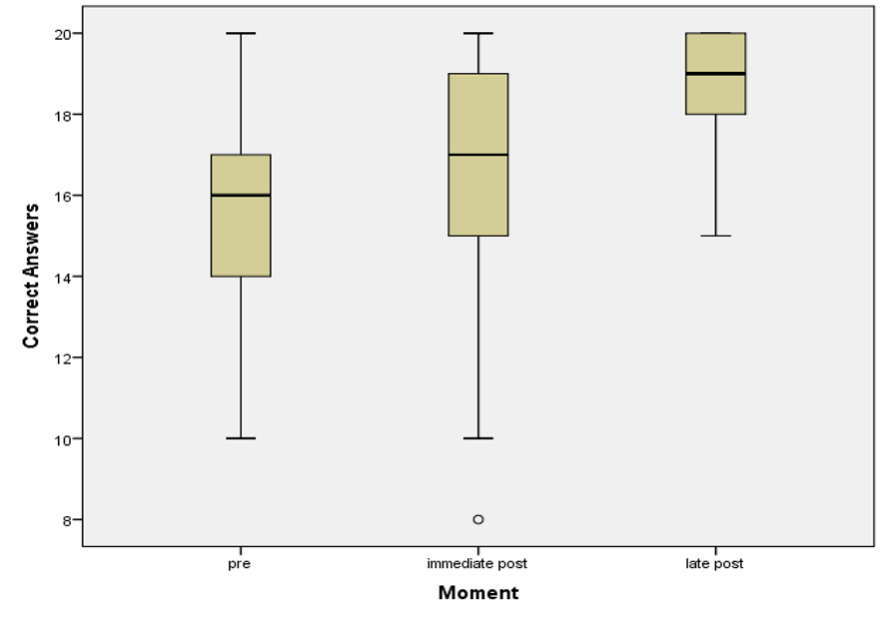
Boxplot graph of the total correct answers per participant at each questionnaire application moment

The comparison of the total number of correct responses demonstrated a significant difference across the three moments (*p*<0.001). Pairwise analysis showed that each time significantly differed from the others (pre *vs*. immediate post, *p*=0.001; pre *vs*. later post, *p*<0.001; immediate post *vs*. later post, *p*<0.001). The lowest median score was observed before the training, with the highest occurring six months after its completion (Figure 2).

**Figure 3:**
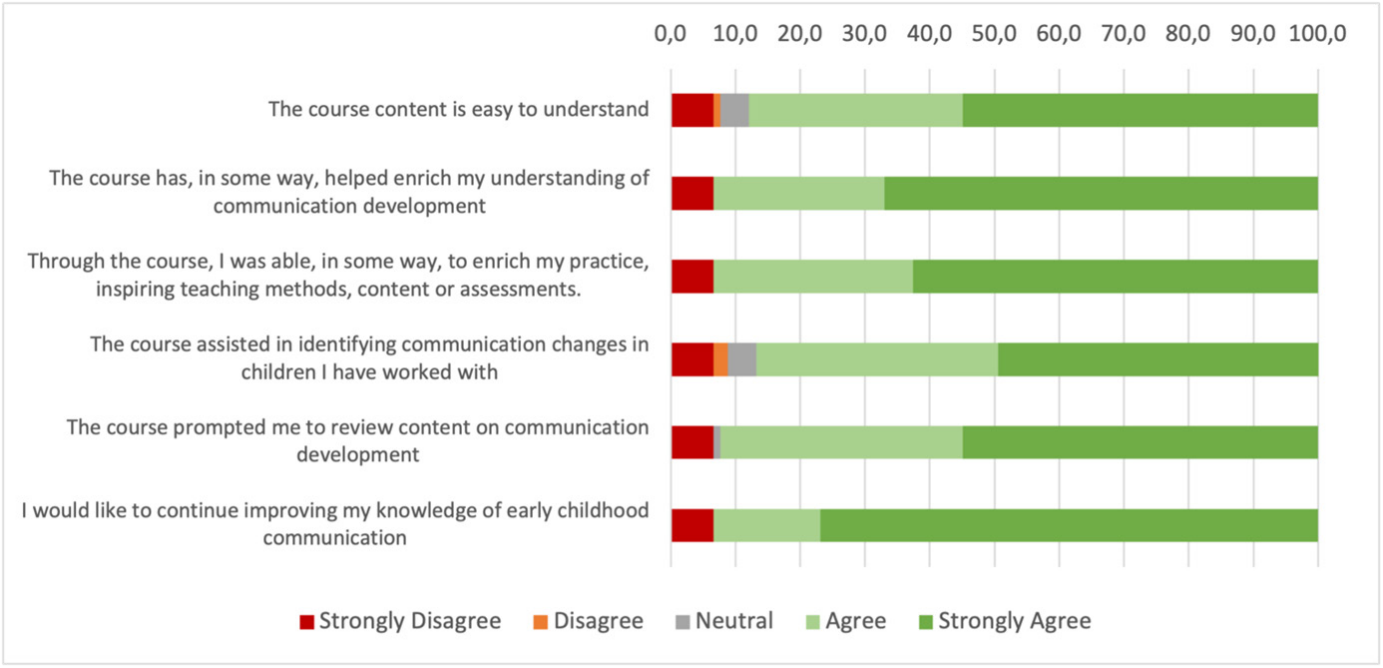
Graph of the frequency distribution of the course perception questionnaire responses

Regarding the participants’ perceptions of the course, most items received more than 90% agreement (responses of agree and strongly agree). Exceptions were noted concerning the ease of understanding the content and the course’s assistance in identifying changes; however, even for these, neutral or disagreeing responses did not exceed 15% (Figure 3).

## DISCUSSION

This study aimed to analyze whether a training course on the development of communication in early childhood positively impacts the knowledge of early childhood education professionals and to verify the participants’ perception of this course.

The comparison across three moments revealed that participants' total number of correct answers increased from pre- to immediate post-training and from immediate post-training to late post-training. These findings indicate that the participants absorbed the information presented. Notably, at the pre- and immediate post-training stages, the distribution of responses displayed greater variability, whereas, in the late post-training stage, the scores were more consistently high. This pattern suggests that the group of participants became more uniform in their response profile over time.

Information retention level is linked to individual engagement and its application frequency. Content is retained in long-term memory only when it is meaningful and regularly utilized. Hence, active involvement with the learned information is crucial for its long-term retention^(15,16)^. We propose that the reason for the increase in correct answers from the immediate to the late post-training is that the professionals reviewed the content after completing the training and applied it in their practice, especially since both groups were reassessed in 2022 – the year when in-person classes in early childhood education resumed.

Although the participants’ motivation was not directly measured, their active engagement with the course material can be inferred. Despite none of the participants having prior training in communication development, their improved performance in the late post-training phase indicates the effectiveness of the training in broadening their knowledge on the subject, even six months after completing the course.

Contrary to previous studies that highlighted the limited knowledge of early childhood education professionals regarding communicative aspects in early childhood^(8,17,18)^, this study found that, from the pre-training stage, the total correct answers exceeded 50%. This divergence may be attributed to our participants proactively enrolling in the course and choosing to participate in the study.

To gain a more comprehensive understanding of potential changes in the participants’ knowledge, we analyzed each questionnaire item, which revealed a consistent increase in the percentage of correct responses for most items. This confirms that participants not only acquired a better understanding of the course content immediately after its conclusion but also retained this enhanced understanding six months later.

For items that did not follow the pattern of gradual increase – specifically, items 2 (*If a child does not speak, they cannot understand what is said to them*), 3 (*Symbolic play aids in language development*), and 11 (*A child spending most of the day with a smartphone, tablet, or computer learns to speak better*), the correct answers decreased from pre- to immediate post-training but increased beyond the pre-training level in the late post-training. Given that the decrease in correct answers was minor (≤5%), the fluctuation in responses across the moments likely reflects a temporary adjustment in knowledge.

Items 16 (*When asking a child a question, it is essential to allow them time to answer before assisting them*) and 17 (*Deaf children cannot attend school*) maintained the same percentage of correct answers from pre-training to immediate post-training but reached the peak in correct answers at the late post-training. This oscillation also appears to be a temporary adjustment in knowledge. It is worth noting that these items initially had correct response rates >95%, rendering the stagnation at the first two moments relatively insignificant.

Items 1 (*A child born deaf cannot develop oral language normally*) and 10 (*Every child with hearing impairment will have significant challenges in understanding spoken words*) had accuracies <50% at the pre-training phase, yet exhibited a gradual increase in correct responses at both the immediate post-training and late post-training stages. These items—focused on the theme of hearing loss—imply that, initially, these professionals possessed limited knowledge on this topic. However, the trend of increasing correct answers suggests that the training provided was effective, enhancing understanding, as evidenced by the immediate post-training accuracy >70% – an approximate 30% improvement from the baseline assessment. Given that educators are key in identifying acquired or manifesting late hearing loss and integrating children with hearing loss into the educational environment^(19)^, the findings from this study underscore the value of equipping them with quality training.

The lowest percentage of correct responses was observed in item 15 (*Children under one year of age generally repeat words when asked*). It recorded accuracies <50% in the first two stages, but in the late post-training stage, it exceeded 70%. Although it was the item with the lowest success rate across all stages, it noted the largest increase in correct answers. Initially, the responses likely stemmed from common sense; however, after the course, the professionals were able to refine their understanding of the topic. This subject involves recognizing how children communicate in the first year of life and the role that repetition (or imitation) plays in communication development. Similar to other items, the increase in correct answers between the post-training stages likely reflects an adjustment in knowledge about the theme, which, along with observation and professional practice, aided its consolidation.

In short, this analysis of the items aimed to ascertain if there were specific topics that posed particular challenges for the participants. The emphasized items do not display a clear pattern but suggest that in future training sessions with such professionals, it might be beneficial to focus more on themes about the normal development of hearing and language. This is supported both by the patterns of responses to the questions and by recent evidence indicating that the quality of professional training for educators positively impacts the linguistic development of children^(20)^.

One aspect to consider is the time interval before the late post-training assessment. Due to the study’s design and its completion timeframe, we opted for 6 months; however, conducting the questionnaire after a longer interval could reveal a different response pattern. In a similar study involving community health workers on childhood hearing, a decline in knowledge was observed 15 months after training completion. This highlights the need for ongoing education^(21)^.

Although it was not possible to gather data on how this shift in knowledge affects professional performance, these findings verify that collaborations between speech-language pathologists and educators, through communication-focused training, can yield significant benefits and potentially influence language development^(4,22,23)^. Such initiatives are viable not only within Speech-Language Pathology in Educational Environment but also as health promotion strategies in school settings^(4)^. This exchange of knowledge enables educational professionals to gain specific skills in language development^(22-24)^.

The National Common Core Curriculum (BNCC) highlights ongoing training for teachers as a cornerstone of early childhood education, emphasizing the need for teachers to stay updated and trained to foster pedagogical practices^(25)^. Moreover, in the aftermath of the COVID-19 pandemic, with the observed impacts of social distancing on child development showcasing declines in communicative and social skills^(26)^, it becomes crucial for educators to continuously update their knowledge to implement the most effective pedagogical approaches in their classrooms.

In Brazil, there is a limited body of research on this topic. Teachers often lack a thorough understanding of communication development and, despite acknowledging its significance for academic learning, feel unprepared to address its implications^(27)^. While our findings focus on professional knowledge, it is worth noting that over 85% of the participants found the course straightforward, beneficial to their professional growth, and motivating towards further education. These results attest to the efficacy of this training model as a strategy for collectively striving for the comprehensive development of children’s communicative skills^(28,29)^. It is also significant that the course was conducted entirely online, suggesting that tele-education can be a supportive tool in professional training within the nexus of Speech-Language Pathology in Educational Environment^(30)^.

As for the study’s limitations, it is possible that participants learned about the research predominantly through social media, which might have limited the dissemination’s reach while attracting a highly interested and motivated sample group. Employing a questionnaire that categorizes responses as either true or false presents another analytical challenge, although the inclusion of an “I don't know” option was intended to reduce guesswork. A further constraint was our inability to assess how the acquisition of new knowledge translated into pedagogical practice changes. Nonetheless, we plan to extend the research to involve direct interactions with educators and their students.

Despite the challenging conditions posed by the COVID-19 pandemic, the findings suggest that the training course’s content and format are effective in broadening these professionals’ knowledge base. Therefore, it can be deduced that offering such courses may enhance educators’ ongoing education. Future studies should explore whether this broadened knowledge base translates into identifying children with potential communication disorders and employing strategies that support communication development in educational settings.

## CONCLUSION

The participants showed an increase in the number of correct responses on the questionnaire both before and after the training course, indicating enhanced knowledge regarding communication development in early childhood. This improvement was noted immediately following the course’s completion and persisted six months later. Furthermore, participants’ evaluations of the course revealed a high level of satisfaction. These findings imply that professional training serves as an effective means to improve the knowledge base of this group.
