# Supplementary material for: Does a training course on early childhood communication have an impact on the knowledge of early childhood education professionals?
Source: Codas. 2024 Jun 14;36(4):e20230192. doi: 10.1590/2317-1782/20242023192en (PMC11189147; doi:10.1590/2317-1782/20242023192en)
Supplement: Translation

# Translation: Um curso de capacitação sobre comunicação na primeira infância impacta no conhecimento de profissionais da educação infantil?

**DOI:** 10.1590/2317-1782/20242023192pt

**Authors:** Hellen Tatyanne da Silva Barbosa, Vanessa Giacchini, Eliene Silva Araújo, Ana Manhani Cáceres-Assenço

**Affiliations:** 1 Laboratório de Desenvolvimento da Linguagem, Departamento de Fonoaudiologia, Centro de Ciências da Saúde, Universidade Federal do Rio Grande do Norte – UFRN - Natal (RN), Brasil.

**Keywords:** Capacitação, Educação Infantil, Retenção (Psicologia), Desenvolvimento da Linguagem, Transtornos da Comunicação

## Abstract

**Objetivo:**

Analisar se um curso de capacitação sobre o desenvolvimento da comunicação na primeira infância tem impacto positivo no conhecimento de profissionais da educação infantil e verificar a percepção dos participantes sobre o curso.

**Método:**

Estudo longitudinal conduzido em ambiente virtual entre setembro de 2021 e dezembro de 2022. No total, participaram 91 profissionais da educação infantil que concluíram um curso de capacitação. O curso consistiu em três módulos sobre desenvolvimento da comunicação na primeira infância, ofertados por meio da plataforma Google Classroom, com uma carga horária total de 50 horas distribuídas ao longo de quatro meses. Os participantes responderam um questionário composto por 20 itens relacionados aos temas abordados antes de iniciar o curso, imediatamente após a conclusão e seis meses após o término. Para cada questão respondida corretamente foi atribuído 1 ponto. A análise dos dados foi conduzida por meio de uma abordagem descritiva e inferencial, sendo a comparação do total de acertos nos três momentos realizada por meio da ANOVA de Friedman, com nível de significância de 5%.

**Resultados:**

Tanto a análise dos acertos de cada item do questionário, quanto a pontuação geral revelou aumento gradual entre os três momentos. A percepção dos participantes sobre o curso teve alto índice de satisfação.

**Conclusão:**

Os participantes demonstraram aumento no número de acertos no questionário nos momentos pré e pós curso de capacitação, o que sugere maior conhecimento sobre o desenvolvimento da comunicação na primeira infância tanto imediatamente após sua conclusão, quanto após seis meses.

## INTRODUÇÃO

A Fonoaudiologia começou a se desenvolver na década de 1920. Nessa época, aqueles que exerciam atividades semelhantes com as do fonoaudiólogo moderno tinham formação relacionada à educação, evidenciando uma ligação entre a profissão e o ensino^(1)^. Somente na década de 1980, com a regulamentação da profissão, a escola foi legalmente reconhecida como um local de atuação para o fonoaudiólogo. Atualmente, a Fonoaudiologia Escolar não se limita apenas a triagens, orientações e encaminhamentos, mas também engloba a participação ativa no processo educacional e inclui atuação com pais, professores, alunos e outros profissionais que compõem a equipe da escola^(2,3)^.

Se por um lado os fonoaudiólogos são profissionais especializados em lidar com os transtornos da comunicação, os profissionais da educação infantil são fundamentais para criar um ambiente educacional adequado à estimulação do desenvolvimento da linguagem e da comunicação nas crianças^(4)^. Assim, a parceria entre a Fonoaudiologia e a Educação pode ser um elo estratégico na promoção do desenvolvimento da comunicação na primeira infância.

Durante os primeiros cinco anos de vida de uma criança, o desenvolvimento ocorre de forma intensa. Habilidades emocionais, sociais e cognitivas são adquiridas e integradas ao cotidiano, especialmente quando se trata do contexto escolar^(5)^. Assim, a escola não se restringe ao aprendizado de conteúdos, mas favorece o desenvolvimento de habilidades e competências. Dentre estas podemos destacar as habilidades de linguagem, uma vez que a variedade de interlocutores é fundamental para a capacidade comunicativa^(6)^.

Fora da família, o educador é o agente mais influente no desenvolvimento de uma criança. Por meio de seu contato diário, ele pode ser o primeiro a perceber dificuldades na comunicação e na aprendizagem^(7,8)^ e os efeitos de sua atuação vão muito além da capacidade de uma criança ler e escrever^(9,10)^. No entanto, para que os educadores possam cumprir mais essa importante tarefa, é necessário que possuam um conhecimento sólido sobre o desenvolvimento da comunicação na primeira infância. Na prática, muitos educadores apresentam dificuldades em identificar e atuar adequadamente em situações que envolvem a comunicação infantil^(11,12)^.

O fonoaudiólogo, com sua formação especializada, pode contribuir nos processos de formação continuada e aprimoramento profissional com educadores. Esta colaboração pode favorecer que os educadores aprimorem sua atuação diante de desafios na comunicação das crianças, promovendo um ambiente de aprendizagem mais inclusivo e estimulante^(13)^. Para se assegurar que a formação continuada se traduza em conhecimento prático, é necessário mensurar sua eficácia, ou seja, a diferença entre o conhecimento antes e após o curso de capacitação. No entanto, compreender se essa mudança ocorre não é suficiente; é necessário verificar a retenção desse conhecimento pelo profissional por um período prolongado após sua conclusão.

Este trabalho teve como objetivos analisar se um curso de capacitação sobre o desenvolvimento da comunicação na primeira infância tem impacto positivo no conhecimento de profissionais da educação infantil, e verificar a percepção dos participantes sobre o curso.

## MÉTODO

O estudo foi aprovado pelo Comitê de Ética em Pesquisa vinculado ao Hospital Universitário Onofre Lopes da Universidade Federal do Rio Grande do Norte, sob protocolo 4.955.205. Os participantes receberam esclarecimentos sobre os objetivos e procedimentos da pesquisa e foram convidados a indicar seu aceite em um termo de consentimento livre e esclarecido, de forma virtual.

A coleta de dados ocorreu entre setembro de 2021 e dezembro de 2022. A amostra foi obtida por conveniência a partir dos inscritos em um curso de capacitação sobre desenvolvimento da comunicação na primeira infância. Esse curso foi divulgado em redes sociais do laboratório de pesquisa ao qual está vinculado e da Universidade. No total, participaram 91 profissionais da educação que realizaram o referido curso de capacitação. Como critérios de inclusão do estudo, foram adotados ser profissional da educação e concluir o curso de capacitação. O único critério de exclusão considerado foi não atuar na educação infantil. As Tabelas 1 e 2 apresentam as características sociodemográficas e o perfil de atuação profissional dos participantes. Vale ressaltar que um número considerável de participantes atua tanto na educação infantil, quanto em outros níveis de ensino.

**Tabela 1:** Distribuição de frequências das variáveis sociodemográficas dos participantes

**Variável**		**Frequência**	**%**
Sexo	Feminino	86	94,5
	Masculino	5	5,5
Idade	Até 24 anos	4	4,4
	De 25 a 29 anos	15	16,5
	De 30 a 34 anos	14	15,4
	De 35 a 39 anos	21	23,1
	De 40 a 44 anos	19	20,9
	De 45 a 49 anos	11	12,1
	De 50 a 54 anos	5	5,5
	55 anos ou mais	2	2,2
Nível máximo de escolaridade	Ensino Médio	1	1,1
	Ensino Superior Completo - Pedagogia	19	20,9
	Ensino Superior Completo - outras Licenciaturas	9	9,9
	Especialização	55	59,8
	Mestrado (acadêmico ou profissionalizante)	3	3,3
	Doutorado	1	1,1
	Outros*	4	4,4
Classificação socioeconômica	A1	10	11,0
	B1	9	9,9
	B2	36	39,6
	C1	23	25,3
	C2	12	13,2
	D-E	1	1,1
Localidade	Natal	41	45,1
	Outras cidades do RN	47	51,6
	Outros estados	3	3,3

* Incluem os profissionais que possuem magistério ou estão cursando o ensino superior

**Tabela 2:** Distribuição de frequências das variáveis relacionadas à atuação profissional dos participantes

**Variável**		**Frequência**	**%**
Atuação como profissional de Educação	01 a 03 anos	17	18,7
	04 a 05 anos	13	14,3
	06 a 10 anos	29	31,9
	11 a 15 anos	15	16,5
	14 a 20 anos	10	11,0
	Mais de 20 anos	7	7,7
Atuação na área da Educação Infantil	01 a 03 anos	25	27,5
	04 a 05 anos	11	12,1
	06 a 10 anos	32	35,2
	11 a 15 anos	10	11,0
	14 a 20 anos	9	9,9
	Mais de 20 anos	2	2,2
	Não se aplica	2	2,2
Nível de ensino com que atua*	CEMEI	51	56,0
	Creche	37	40,7
	Ensino Fundamental I	39	42,9
	Ensino Fundamental II	8	8,8
	Ensino Médio	4	4,4
	Educação de Jovens e Adultos	1	1,1
Função	Professor	57	62,7
	Coordenador Pedagógico	14	15,3
	Auxiliar de Desenvolvimento Infantil	9	9,9
	Estagiária	3	3,3
	Gestor	3	3,3
	Apoio pedagógico	1	1,1
	Professor do Atendimento Educacional Especializado (AEE)	1	1,1
	Professora da Sala de Recursos Multifuncionais	1	1,1
	Atendente terapêutico	1	1,1
	No momento não desempenho	1	1,1

* Considerando que muitos participantes atuam em mais de uma instituição de ensino e que em muitos casos esta atuação ocorre em diferentes níveis, foi contabilizado o número de vezes que cada nível foi indicado

### Contexto

O curso de capacitação foi realizado por meio da plataforma virtual *Google Classroom*, com uma carga horária total de 50 horas, distribuídas ao longo de quatro meses. Ele foi desenvolvido em um projeto de extensão universitária voltado para profissionais da educação e seu conteúdo programático abrangeu o conhecimento sobre o desenvolvimento típico e atípico da comunicação na primeira infância. Os conteúdos foram divididos em três módulos: (1) Aquisição e desenvolvimento da linguagem oral; (2) Os transtornos da comunicação comuns na primeira infância; (3) Estratégias para estimular a comunicação no contexto educacional. As aulas foram ministradas por docentes universitários do curso de Fonoaudiologia, cuja área de atuação envolve linguagem ou audição na infância. Cada módulo era composto por videoaulas com cerca de 30 minutos cada e ao final de cada aula era necessário que o participante respondesse a cinco questões de múltipla escolha sobre o tema (Figura 1).

**Figura 1:** Detalhamento das características do curso de capacitação

Cada participante acessou o material de forma individualizada e definiu seu próprio ritmo de estudos, porém conduzidos por prazos pré-estabelecidos. Os módulos foram liberados num intervalo de quatro semanas, sendo definido o prazo total de 16 semanas para conclusão do curso. Vale destacar que foram ofertadas duas turmas do curso durante o período do estudo, ambas com o mesmo conteúdo e carga horária. Dentre os 383 inscritos no curso, 203 (53,0%) acessaram as aulas e destes 123 (32,1%) concluíram todas as atividades no prazo. Destes ainda foi preciso excluir 32 devido ao critério de exclusão ou por não consentir em participar.

### Medidas de saída

Para mensurar o conhecimento a respeito do desenvolvimento da comunicação na primeira infância foi elaborado um questionário, tendo como referência o instrumento utilizado em outros estudos que analisaram a eficácia de cursos de capacitação para profissionais da atenção primária à saúde^(14)^. Manteve-se a estrutura de 20 itens, as opções de resposta e a forma de pontuação, no entanto, foi realizada adaptação das afirmativas de forma que contemplasse exclusivamente os conteúdos abordados no curso de capacitação.

Ao responder o questionário, o participante teve que assinalar cada item como “verdadeiro” ou “falso”. Além disso, foi incluída a opção “não sei” para minimizar a ocorrência de respostas ao acaso, permitindo ao participante indicar quando não tinha certeza sobre determinado item. Para cada questão respondida corretamente, foi atribuído um ponto, assim a pontuação poderia variar entre zero e 20.

O questionário foi disponibilizado online na plataforma *Google Forms,* e sua aplicação ocorreu em três momentos distintos: (1) antes de iniciar o curso, (2) imediatamente após sua conclusão e (3) seis meses após a conclusão.

Para avaliar o quanto cada participante considerou o conteúdo do curso relevante para sua prática, elaboramos um questionário sobre a percepção em relação ao curso. O questionário foi composto por seis afirmativas que deveriam ser respondidas em uma escala Likert de cinco pontos com as seguintes possibilidades de resposta: discordo totalmente, discordo, neutro, concordo e concordo totalmente. Ressalta-se que sua aplicação ocorreu imediatamente após a conclusão do curso e a análise considerou a porcentagem de participantes que indicaram cada possibilidade de resposta por afirmativa.

### Análise dos dados

A análise do questionário de conhecimento considerou tanto a porcentagem de respostas corretas em cada item do questionário, quanto a porcentagem total de acertos por participante. Para verificar o nível de eficácia e retenção das informações abordadas na capacitação, realizamos a comparação dos acertos nos três momentos de aplicação do questionário. A análise estatística foi realizada por meio do software *Statistical Package for the Social Sciences* (SPSS), versão 24. Utilizamos análise descritiva para examinar a frequência de respostas corretas, e a análise inferencial foi realizada por meio da ANOVA de Friedman com comparações múltiplas e um nível de significância de 5%.

## RESULTADOS

A análise de cada item do questionário revelou um padrão de aumento gradual da porcentagem de acertos ao longo dos três momentos, com poucas exceções (itens 2, 3, 11, 16 e 17). A análise de cada momento indicou que no pré capacitação três itens tiveram menos de 50% (itens 1, 10 e 15), no pós imediato apenas um item teve menos de 50% de acertos (item 15), enquanto no momento pós tardio a menor frequência foi 71,4% (Tabela 3).

**Tabela 3:** Frequência de acertos, em porcentagem, para cada item do questionário sobre o conhecimento do desenvolvimento da comunicação

**Item**	**Pré**	**Pós imediato**	**Pós tardio**
1. A criança que nasce surda não pode desenvolver linguagem oral normalmente.	46,2	73,6	83,5
2. Se a criança ainda não fala, ela não consegue entender o que escuta	91,2	86,8	98,9
3. A brincadeira simbólica favorece o desenvolvimento da linguagem	100,0	98,9	100,0
4. Uma criança com problema auditivo pode apresentar problemas de comportamento em consequência da dificuldade em ouvir.	87,9	91,2	95,6
5. Durante os primeiros anos de vida as crianças podem apresentar sintomas de gagueira.	68,1	91,2	100,0
6. Conhecer muitas palavras ajuda as crianças a prestarem atenção aos sons que as compõem	79,1	83,5	91,2
7. A orelha humana é capaz de ouvir sons de frequências graves, médias e agudas	73,6	87,9	100,0
8. É normal que uma criança ainda não combine palavras aos 2 anos	54,9	64,8	83,5
9. Quando uma criança tem dificuldades para se comunicar pela fala, ela tem menos capacidade para aprender	71,4	89,0	98,9
10. Toda criança deficiente auditiva terá muita dificuldade para ouvir o que as pessoas falam	48,4	62,6	75,8
11. A criança que brinca com celular, tablet ou computador na maior parte do dia aprende a falar melhor	93,4	90,1	98,9
12. Rimas e aliterações favorecem o desenvolvimento da linguagem	92,3	95,6	98,9
13. Infecções de ouvido não tratadas podem causar deficiência auditiva	82,4	92,3	98,9
14. Ao conversar com uma criança que está com dificuldades para se comunicar é interessante falar rápido e com frases longas e bem elaboradas	94,5	97,8	98,9
15. Crianças menores de um ano em geral repetem palavras quando solicitadas	37,4	39,6	71,4
16. Quando perguntamos algo para uma criança é preciso dar um tempo para ela responder antes de ajudarmos com a resposta	96,7	96,7	100,0
17. Crianças surdas não podem ir à escola	98,9	98,9	100,0
18. Aos 5 anos a criança já deveria falar todos os sons da língua de forma clara	54,9	76,9	87,9
19. Uma criança que gagueja pode passar a gagueira para outras crianças de seu convívio	87,9	89,0	100,0
20. Se uma criança fala de forma difícil de compreender aos 4 anos ela precisa passar por uma avaliação com fonoaudiólogo	89,0	96,7	100,00

A comparação do total de acertos revelou diferença significante nos três momentos (p<0,001). A análise par a par indicou que todos os momentos diferiram entre si (pré x pós imediato p=0,001; pré x pós tardio p<0,001; pós imediato x pós tardio p<0,001). A menor mediana foi observada antes da capacitação e a maior seis meses após sua conclusão (Figura 2).

**Figura 2:** Gráfico de boxplot do total de acertos por participante em cada momento de aplicação do questionário

Com relação à percepção dos participantes sobre o curso, a maioria dos itens teve mais de 90% de concordância (respostas concordo e concordo totalmente). As exceções se relacionam aos conteúdos serem de fácil compreensão e o auxílio do curso na identificação de alterações, mas ainda assim as respostas neutras ou discordantes não alcançaram 15% (Figura 3).

**Figura 3:** Gráfico da distribuição de frequência das respostas do questionário de percepção do curso

## DISCUSSÃO

Este estudo teve como objetivos analisar se um curso de capacitação sobre o desenvolvimento da comunicação na primeira infância tem impacto positivo no conhecimento de profissionais da educação infantil e verificar a percepção dos participantes sobre o curso.

A comparação entre os três momentos mostrou que o total de acertos dos participantes aumentou tanto do pré para o pós imediato, quanto do pós imediato para o pós tardio. Esses resultados sugerem que os participantes assimilaram as informações abordadas. Vale destacar que nos momentos pré e pós imediato a distribuição das respostas teve maior variabilidade, enquanto no pós tardio os valores se concentraram próximos ao máximo. Este dado sugere que ao longo dos momentos, o grupo de participantes se tornou mais homogêneo em seu perfil de respostas.

O nível de retenção de uma informação está relacionado com o envolvimento individual e com sua frequência de utilização. Um conteúdo será armazenado memória de longo prazo apenas quando for significativo e utilizado de forma rotineira. Portanto, o engajamento ativo com as informações adquiridas é fundamental para promover sua retenção duradoura^(15,16)^. Nossa hipótese para o aumento de acertos entre o pós imediato e o pós tardio é de que os profissionais revisitaram os conteúdos após a conclusão da capacitação para que pudessem aplicá-los em sua prática, visto que ambas as turmas foram reavaliadas em 2022, ano em que as aulas presenciais haviam sido retomadas na educação infantil.

Ainda que não tenhamos mensurado diretamente a motivação dos participantes, é possível inferir que estes se engajaram com os estudos. Mesmo que nenhum deles possuísse formação prévia sobre o desenvolvimento da comunicação, o melhor desempenho na etapa pós tardio mostrou que a capacitação foi eficaz para ampliar o conhecimento sobre o tema, mesmo após um semestre de conclusão do curso.

Apesar de estudos anteriores indicarem que o conhecimento de professores de educação infantil é restrito quanto aos aspectos comunicativos na primeira infância^(8,17,18)^, neste estudo, desde o momento pré-capacitação, o índice de acertos total se concentrou acima dos 50%. É possível que este padrão tenha sido observado porque, diferente de outros estudos, os participantes tiveram a iniciativa de se inscrever no curso e optaram por colaborar com o estudo.

Para compreender melhor as possíveis mudanças no conhecimento dos participantes, consideramos cada item do questionário, o que nos permitiu identificar que para a maioria deles houve um aumento gradual da porcentagem de acertos. Novamente, isso indica que imediatamente após a conclusão do curso os participantes demonstraram conhecer melhor os temas abordados e que mantiveram esse padrão de melhora após seis meses de conclusão.

Dentre os itens que fugiram do padrão de aumento gradual, os itens 2 (*Se a criança ainda não fala, ela não consegue entender o que escuta*), 3 (*A brincadeira simbólica favorece o desenvolvimento da linguagem*) e 11 (*A criança que brinca com celular, tablet ou computador na maior parte do dia aprende a falar melhor*) tiveram redução dos acertos do pré para o pós imediato, mas no pós tardio superaram os acertos do pré. Considerando que a redução da porcentagem de acertos foi discreta, pois não alcançou 5%, a oscilação das respostas entre os momentos pode refletir apenas um período de reacomodação do conhecimento.

Já os itens 16 (*Quando perguntamos algo para uma criança é preciso dar um tempo para ela responder antes de ajudarmos com a resposta*) e 17 (*Crianças surdas não podem ir à escola*) mantiveram a mesma porcentagem no pré capacitação e pós imediato, mas alcançaram índice máximo de acerto no pós tardio. Este perfil de oscilação também nos parece refletir apenas uma reacomodação do conhecimento. Vale destacar que estes itens já apresentaram índices de acerto superiores a 95%, portanto esta estagnação nos dois primeiros momentos não nos parece significativa.

Os itens 1 (*A criança que nasce surda não pode desenvolver linguagem oral normalmente*) e 10 (*Toda criança deficiente auditiva terá muita dificuldade para ouvir o que as pessoas falam*) tiveram menos que 50% de acerto no momento pré capacitação, mas demonstraram aumento gradual dos acertos nos momentos pós imediato e pós tardio. Ambos os itens estão relacionados ao tema da perda auditiva, o que sugere que, previamente ao curso, estes profissionais dispunham de informações restritas a esse respeito. Entretanto, o padrão crescente de acertos indica que a capacitação forneceu informações suficientes para sua compreensão, afinal os acertos no pós imediato alcançaram mais de 70%, o que representa um aumento de quase 30% em relação ao primeiro momento. Ao considerar que o educador possui um papel fundamental na identificação de perdas auditivas adquiridas ou de manifestação tardia e na inclusão da criança com perda auditiva no ambiente escolar^(19)^, os dados obtidos no presente estudo, reforçam a importância de capacitá-los para uma atuação de qualidade.

A menor porcentagem de acertos foi observada no item 15 (*Crianças menores de um ano em geral repetem palavras quando solicitadas*). Nos dois primeiros momentos, ele teve menos de 50% de acerto, mas no pós tardio alcançou mais de 70%. Apesar de ser o item com menor índice de acertos em todos os momentos, ele foi o item que apresentou maior aumento de acertos. No momento pré-capacitação é bastante provável que as respostas tenham sido baseadas no senso comum, porém após o curso os profissionais tiveram condições de rever seus conceitos sobre o tema. Tal conteúdo envolve conhecer como uma criança é capaz de se comunicar no primeiro ano de vida e também compreender a função que a repetição (ou imitação) desempenha no desenvolvimento da comunicação. De forma semelhante aos demais índices, o aumento dos acertos entre os momentos pós-capacitação pode refletir uma reacomodação dos saberes sobre o tema, que associado à observação e à prática profissional, favoreceram sua consolidação.

Em síntese, esta análise dos itens buscou identificar se haveria algum assunto com maior fragilidade para os participantes. Os itens destacados não demonstram um padrão claro, porém indicam que em novas oportunidades de capacitação com tais profissionais pode ser útil enfatizar temas relacionados ao desenvolvimento típico da audição e da linguagem. Tal sugestão se justifica tanto pelo padrão de resposta às questões, quanto pelas evidências recentes de que a qualidade da capacitação profissional dos educadores impacta de forma positiva no desenvolvimento linguístico das crianças^(20)^.

Um ponto que merece ser considerado se refere ao tempo de intervalo para o pós tardio. Devido à natureza do estudo e o prazo para sua conclusão, adotamos um intervalo de 6 meses, porém uma nova aplicação do questionário com um intervalo maior poderia revelar um padrão diferente de resposta. Em um estudo semelhante realizado com agentes comunitários de saúde sobre audição na infância, após 15 meses de conclusão da capacitação os participantes apresentaram redução no conhecimento. As autoras apontam que este achado reforça a necessidade de uma formação continuada^(21)^.

Ainda que não tenha sido possível obter dados sobre a repercussão dessa mudança de conhecimentos na atuação profissional, estes resultados confirmam que a parceria entre fonoaudiólogos e educadores, por meio da capacitação sobre aspectos da comunicação, pode trazer benefícios significativos e com potencial impacto no desenvolvimento da linguagem^(4,22,23)^. Este tipo de atuação se configura não apenas como uma possibilidade do fazer da Fonoaudiologia Educacional, mas também como uma estratégia de promoção de saúde no contexto escolar^(4)^. A troca de conhecimentos permite que os profissionais de educação adquiram competências específicas relacionadas ao desenvolvimento da linguagem^(22-24)^.

A formação continuada para os professores é apontada pela Base Nacional Comum Curricular (BNCC) como um dos pilares da educação infantil, pois os professores precisam estar atualizados e capacitados para desenvolver práticas pedagógicas^(25)^. Somado a essa diretriz, no cenário pós pandemia de COVID-19, em que o impacto do distanciamento social no desenvolvimento infantil vem indicando prejuízos em habilidades comunicativas e de socialização^(26)^, é fundamental que os educadores estejam em constante atualização para que possam utilizar as melhores práticas pedagógicas em sala de aula.

A literatura sobre este tema ainda é escassa no Brasil, mas os professores não costumam ter um conhecimento profundo sobre o desenvolvimento da comunicação e, apesar de considerarem sua importância para o aprendizado escolar, não se sentem seguros para lidar com suas repercussões^(27)^. Por mais que nossos resultados estejam centrados no conhecimento dos profissionais, um aspecto que merece destaque se refere à percepção sobre o curso, pois mais de 85% dos participantes consideraram o curso de fácil compreensão, benéfico para sua atuação profissional e instigante a dar continuidade nos estudos. Tal achado reforça que este modelo de capacitação pode ser uma estratégia válida na busca conjunta para o desenvolvimento pleno do potencial de comunicação na infância^(28,29)^. Ainda é interessante pontuar que como o curso foi desenvolvido totalmente em ambiente virtual, esta avaliação positiva indica que a teleducação pode ser aliada no processo de capacitação profissional na parceria entre a Fonoaudiologia e a Educação^(30)^.

Dentre as limitações do estudo podemos considerar que os participantes provavelmente souberam da pesquisa por meio de redes sociais, o que pode ter restringido o alcance da divulgação e, ao mesmo tempo, contribuído para uma amostra com alto índice de interesse e motivação sobre o tema. O fato de termos utilizado um questionário que considera cada item como verdadeiro ou falso também traz limitações à análise da eficácia do curso, porém propusemos a alternativa “não sei” para minimizar respostas aleatórias. Outra limitação consiste em não termos conseguido avaliar o quanto o acesso a novas informações se reverteu em mudanças na prática pedagógica. Porém, pretendemos dar continuidade ao estudo e incluir a atuação direta com os educadores e suas turmas.

Apesar das condições adversas vivenciadas durante do estudo, devido à pandemia de COVID-19, os resultados sugerem que o conteúdo e o formato do curso de capacitação são eficazes para ampliar o conhecimento destes profissionais. Assim, é possível inferir que a oferta deste tipo de curso pode contribuir para a formação continuada dos educadores. Em estudos futuros será interessante investigar se essa ampliação dos conhecimentos reflete na identificação de crianças com possíveis transtornos da comunicação e no uso de estratégias que possam favorecer o desenvolvimento da comunicação em ambiente escolar.

## CONCLUSÃO

Os participantes demonstraram aumento no número de acertos no questionário nos momentos pré e pós curso de capacitação, o que sugere maior conhecimento sobre o desenvolvimento da comunicação na primeira infância. Esta melhora foi observada tanto imediatamente após sua conclusão, quanto após seis meses dela. Além disso, a percepção dos participantes sobre o curso teve alto índice de satisfação. Esses resultados sugerem que a capacitação profissional é uma ferramenta útil para aprimorar o conhecimento desta população.
